# Genetic analysis provides insights into species distribution and population structure in East Atlantic horse mackerel (*Trachurus trachurus* and *T. capensis*)

**DOI:** 10.1111/jfb.14276

**Published:** 2020-02-20

**Authors:** Amy J. E. Healey, Matthew W. Farthing, Francis K. E. Nunoo, Warren M. Potts, Warwick H. H. Sauer, Ilze Skujina, Nathan King, Sophie de Becquevort, Paul W. Shaw, Niall J. McKeown

**Affiliations:** ^1^ Institute of Biological, Environmental and Rural Sciences Aberystwyth University Aberystwyth UK; ^2^ Department of Ichthyology & Fisheries Science Rhodes University Grahamstown South Africa; ^3^ Department of Marine and Fisheries Science University of Ghana Accra Ghana; ^4^ School of Ocean Sciences Bangor University Menai Bridge, Anglesey UK

**Keywords:** biogeography, fish, genetic, species, stock, taxonomy

## Abstract

Two sister species of horse mackerel (*Trachurus trachurus* and *T. capensis*) are described that are intensively harvested in East Atlantic waters. To address long‐standing uncertainties as to their respective geographical ranges, overlap and intraspecific population structure this study combined genetic (mitochondrial DNA and microsatellite) analysis and targeted sampling of the hitherto understudied West African coast. mtDNA revealed two reciprocally monophyletic clades corresponding to each species with interspecies nuclear differentiation supported by *F*_ST_ values. The *T. trachurus* clade was found across the north‐east Atlantic down to Ghana but was absent from Angolan and South African samples. The *T. capensis* clade was found only in South Africa, Angola and a single Ghanaian individual. This pattern suggests that both species may overlap in the waters around Ghana. The potential for cryptic hybridization and/or indiscriminate harvesting of both species in the region is discussed. For *T. capensis* mtDNA supports high gene flow across the Benguela upwelling system, which fits with the species' ecology. The data add to evidence of a lack of significant genetic structure throughout the range of *T. trachurus* though the assumption of demographic panmixia is cautioned against. For both species, resolution of stock recruitment heterogeneity relevant to fishery management, as well as potential hybridization, will require more powerful genomic analyses.

## 1 INTRODUCTION

The horse/jack mackerels are a widely distributed genera of Carangid fish found throughout temperate, tropical and subtropical oceanic and coastal waters (Fricke & Eschmeyer, 2019). Across the temperate waters of the north‐east (NE) Atlantic, Mediterranean and southern Africa two sister species, *Trachurus trachurus* and *Trachurus capensis*, are described based on morphological (Nekrasov, 1976; but see Froese & Pauly, 2001) and genetic (Cardenas *et al*., 2005; Karaiskou *et al*., 2004) characteristics. *T. capensis* (Catelnau, 1861) is associated with the Benguela Upwelling System (BUS) and is most often described as extending from southern Angola, throughout Namibia and into the Eastern Cape of South Africa (Axelsen *et al*., 2004; Barange *et al*., 1998). *T. trachurus* is widely described throughout the NE Atlantic as far north as Norway and Iceland (Knijn *et al*., 1993) as well as the Mediterranean basins (Fischer, 1973). Both species are of great commercial importance and are the most valuable horse mackerel species in these regions (Murta, 2000) as exemplified by the Namibian *T. capensis* fishery, which reports the highest biomass and catch of all fish species landed within the region and is the second highest economic contributor to Namibian fisheries (Kirchner *et al*., 2010).

*T. trachurus* has demonstrated severe reductions in total annual catch with the stocks in the Mediterranean, Alboran and Black seas considered fully exploited, while the NE Atlantic stock is deemed to be overexploited (ICES, 2003, 2005). This has led to *T. trachurus* being listed as “vulnerable” on the IUCN's red list of threatened species (Smith‐Vaniz *et al*., 2015) and added further impetus to align management and biological units for both species to try and ensure fishery sustainability (reviewed in: Abaunza *et al*., 2008a) and maintenance of biodiversity. Genetic studies of *T. trachurus* employing mitochondrial DNA (mtDNA) (Comesaña *et al*., 2008; Karaiskou *et al*., 2003, 2004), allozyme (Cimmaruta *et al*., 2008) and microsatellite (Kasapidis & Magoulas, 2008; Sala‐Bozano *et al*., 2015) markers have typically reported a general lack of genetic differentiation across NE Atlantic and Mediterranean samples. In the absence of robust evidence of population structure the species is managed using regionally designated stocks (ICES, 2003, 2005). Similarly, population genetic studies of *T. capensis* have reported no evidence of population structure (Karaiskou *et al*., 2004; Naish, 1990), with the species currently managed as arbitrarily delineated northern and southern BUS stocks (Hecht, 1990; Naish *et al*., 1991).

Studies to date have focused on either the NE Atlantic/Mediterranean or the south‐west African coast, meaning that *T. trachurus* and *T. capensis* diversity within the Angolan/North African region is poorly resolved. Such a geographical sampling gap also adds ambiguity as to the southern and northern limits of *T. trachurus* and *T. capensis*, respectively. Although the majority of studies cite the distribution of *T. trachurus* as extending only as far south as Senegal and the Cape Verde islands (*e.g.*, Abaunza *et al*., 2008a; Cimmaruta *et al*., 2008; Comesaña *et al*., 2008; Sala‐Bozano *et al*., 2015), others recognize a far wider range, encompassing the west African tropics and ranging as far south as South Africa and southern Mozambique (Boateng, 2019; Koranteng, 2002; Smith‐Vaniz *et al*., 2015). Considering the extensive fishing pressure on *T. capensis* in Namibia, the potential for overlap and thus a cryptic mixed stock with *T. trachurus* must be assessed. Additionally, the available genetic data do not sufficiently test the validity of the currently recognized north and south BUS stocks for *T. capensis*.

To address these fundamental knowledge gaps for both species this study focused on collecting samples of putative *T. trachurus*/*T. capensis* from the understudied west African coast alongside better studied, and thus reference, NE Atlantic/Mediterranean (*T. trachurus*) and South African (*T. capensis*) waters. Genetic analysis of sequence variation at two mtDNA regions and 10 microsatellite loci were used to delineate species and assess population structure within both species. The results provide new insight into the distribution of both species and their eco‐evolutionary dynamics while also providing information as to the relationship between population genetic structure and current management units.

## 2 MATERIALS AND METHODS

### 2.1 Sample acquisition and DNA extraction

**Figure 1:**
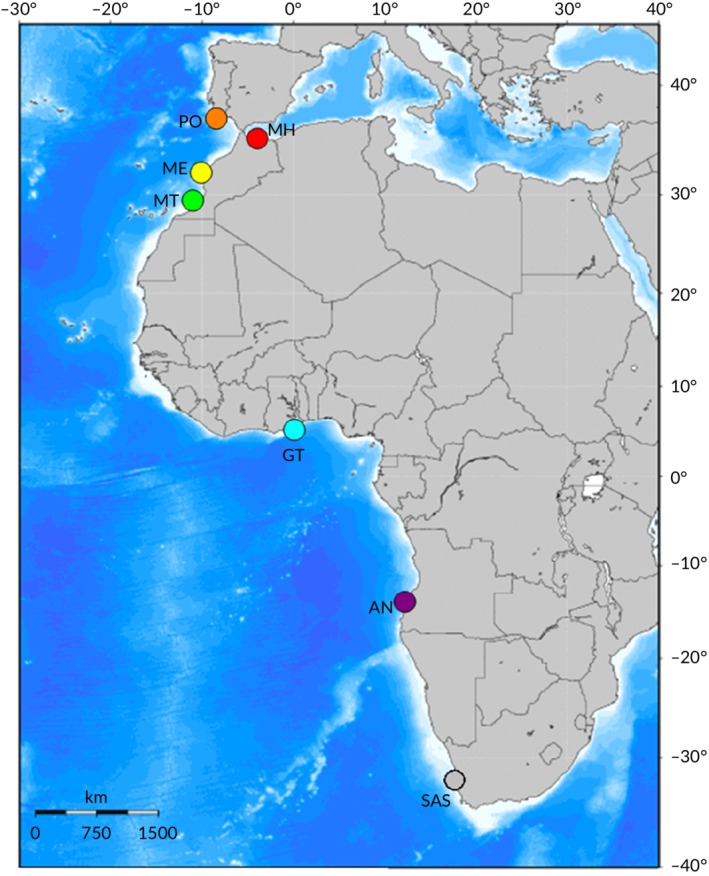
Sampling locations of horse mackerel across the Mediterranean and east Atlantic. MH, Morocco, Al Hoceima; PO, Portugal, Olhao; ME, Morocco, Essaouria; MT, Morrocco, Tiznit; GT, Ghana, Tema; SAS, South Africa, Saldanha

This study complied with all ethical requirements of the *Journal of Fish Biology* and local authorities. No fish were killed or interfered with in any way as part of this study as all samples were in the form of ethanol‐preserved fin clips of adults acquired solely from artisanal fish markets to ensure local providence of individuals. Samples were acquired from locations (Table 1 and Figure 1) across the western Mediterranean basin (Morocco: Al Hoceima), the NE Atlantic (Portugal: Olhao, Morocco: Essaouria and Tiznit), the tropical/subtropical Atlantic coasts of West Africa (Ghana: Tema, Angola: Namibe), and the cool temperate west coast of South Africa (Saldanha) between July and September 2017. Total DNA was extracted using a standard CTAB‐chloroform/isoamyl alcohol method (Winnepenninckx, 1993).

**Table 1:** Sample information and summary indices for mtDNA and microsatellite analysis

Sample	mtDNA		Microsatellite		
Region: Site: Code	Sample *n*, NHap	*h*	Sample *n*, NA, *A*_R_	*H*_*O*_ ‐ *H*_*E*_	*P*HWE
Portugal: Olhao: PO	16, 14	0.983	12, 6.8, 5.5	0.485–0.695	**<0.0001**
Morocco: Al Hoceima: MH	15, 9	0.876	13, 7.5, 5.9	0.504–0.731	**<0.0001**
Morocco: Essaouira: ME	22, 16	0.931	48, 10.8, 6.2	0.539–0.766	**<0.0001**
Morocco: Tiznit: MT	21, 14	0.895	35, 10.7, 6.1	0.544–0.758	**<0.0001**
Ghana: Tema: GT	20, 14	0.916	49, 11.2, 6.3	0.566–0.781	**<0.0001**
Angola: Namibe: AN	9, 9	1	9, 6.3, 5.7	0.691–0.681	0.138
South Africa: Saldanha: SAS	16, 8	0.758	23, 10.1, 6.6	0.619–0.794	**<0.0001**

*Note*: Sample *n* denotes the number of individuals genotyped for each marker type. Code denotes the sample identifier used in Figure 1 and other tables. For mtDNA the number of haplotypes (NHap) and haplotype diversity (*h*) are reported. For microsatellites the number of alleles (NA), allelic richness (*A*_R_, standardized sample size = 9), and observed and expected heterozygosities (*H*_*O*_ and *H*_*E*_, respectively) averaged over loci are reported. *P*HWE are the *P* values from multilocus tests of conformance to Hardy–Weinberg equilibrium.

### 2.2 mtDNA amplification and analysis

Two mtDNA regions were sequenced. A fragment of the cytochrome oxidase subunit 1 (COI) was PCR amplified using the Fish F1 and Fish R1 universal primers (Ward *et al*., 2005) and a fragment of the control region (CR) was PCR amplified using primers (TrachurusCRF2: 5′‐CCAGTCCAACCTGCAAAGGA‐3′ and TrachurusCRR2: 5′‐CACAGGGAGGCGGATACTTG‐3′) designed during this study. Individual PCRs were performed in 20 μl total volumes, containing 50–100 ng template DNA, 2 pmol of each primer, 10 μl of 2XBioMix (Bioline,UK) and 2 μl of ddH_2_O. The PCR thermoprofile was 3 min at 95°C, followed by 35 cycles of 95°C for 30 s, 45 s annealing at 54°C for COI or 52°C for CR amplification and 1 min at 72°C, followed by a final extension stage at 72°C for 3 min. Amplicons were purified and sequenced with respective forward and reverse primers using BIG DYE technology. Sequence identity was confirmed by BLAST [standard database‐nucleotide collection (nr/nt)] and sequences were aligned using the CLUSTALW program (Thompson *et al*., 1994), implemented in BIOEDIT (Hall, 1999).

Phylogenetic relationships among haplotypes were inferred using (a) a minimum spanning network constructed in NETWORK (http://www.fluxus-engineering.com/sharenet.htm); and (b) both maximum likelihood (ML) and Bayesian inference (BI) trees constructed in PHYML (Guindon *et al*., 2010) and MrBayes (Ronquist & Huelsenbeck, 2003), respectively. ML and BI trees were generated using the optimal substitution model (HKY + G) identified by ModelTest (Posada & Crandall, 1998). BI trees were run using three heated and one cold chain over 5,000,000 generations, with the Markov chain sampled at 1000 generation intervals, and the first 15% of trees discarded as burn‐in. BI posterior probabilities were calculated over the 5,000,000 generation run and ML support was calculated based on measures of bootstrap (BS), inferred after 1000 replicates.

ARLEQUINv3.5.2.2 (Excoffier & Lischer, 2010) was used to estimate indices of haplotype diversity (*h*) and to quantify differentiation between pairs of samples, using pairwise Φ_ST_, with significances assessed by 10,000 permutations.

Fu's *F*s (Fu, 1997) and Tajima's *D* (Tajima, 1989) tests were used to test for deviations from mutation‐drift equilibrium that could be attributed to selection and/or population size changes. Mismatch distributions, the frequency distributions of pairwise differences between haplotypes within a sample and simulated distributions under a model of demographic expansion, were compared using the sum of squared deviations (SSD) as a test statistic with significance assessed after 10,000 bootstrap replications. The timing of expansions (*T*) was estimated using the formula *T* = *τ*/2*u* (Rogers & Harpending, 1992). IMa2 (Hey, 2009) was used to estimate divergence times between groups with analyses being run for 1,000,000 burn‐in generations and >5,000,000 sampling generations so that the minimum ESS across parameters was >50 (Hey & Nielsen, 2004). Mismatch and IMa2 analyses were performed using both a universal divergence rate for marine fish species mtDNA of 1.5% per million years (Bermingham *et al*., 1997) and a fossil calibrated *Trachurus* specific divergence rate of 0.14% per million years proposed by Cardenas *et al*. (2005).

### 2.3 Microsatellite amplification and analysis

Individuals were analysed at 10 microsatellite loci, four of which (Tt29, Tt48, Tt62, Tt113) were developed for *T. trachurus* (Kasapidis & Magoulas, 2008) and six (TmurA101, TmurA104, TmurA115, TmurB104, TmurB116, TmurC4) for *T. murphyi* (Canales‐Aguirre *et al*., 2010). Loci were individually PCR amplified in 10 μl reaction volumes containing 50–100 ng template DNA, 1 pmol of each primer, 5 μl of 2XBioMix (Bioline, UK) and 1 μl of ddH_2_O. The PCR thermoprofile for Tt29, Tt48, Tt62 and Tt113 amplification was 95°C for 3 min, followed by 45 cycles of 95°C for 30 s, 30 s annealing at 54°C (for Tt29, Tt48, Tt113) or 52°C (for Tt62) and a 30 s extension step at 72°C, followed by a final extension step of 72°C for 3 min. The other six microsatellite loci (TmurA101, TmurA104, TmurA115, TmurB104, TmurB116, TmurC4) were PCR amplified with the following thermoprofile: 94°C for 3 min, followed by 35 cycles of 94°C for 40 s, 30 s annealing at 57°C (TmurA104, TmurA115, TmurB104, TmurB116, TmurC4) or 52°C (TmurA101) and 30s at 72°C, with a final extension step of 72°C for 5 min. Amplicons were separated on an Applied Biosystems 3500 and alleles were inferred using Peak Scanner software (Applied Biosystems).

Genetic variation within samples was characterized using the number of alleles (*N*_A_), allelic richness (*A*_R_), observed heterozygosity (*H*_O_) and expected heterozygosity (*H*_E_), all calculated using GENALEX 6.2 (Peakall & Smouse, 2006). GENALEX was also used to test for genotype frequency conformance to Hardy–Weinberg expectations (HWE) and genotypic linkage equilibrium between pairs of loci. As locus × sample tests of HWE yielded a large number of deviations due to heterozygote deficits, the software FreeNA (Chapuis & Estoup, 2006) was used to estimate frequencies of null alleles. Genetic differentiation among samples was quantified using *F*_ST_ values with significance assessed following 9999 permutations in GENALEX with pairwise *F*_ST_ visualized using a principal coordinate analysis (PCoA). Global and pairwise *F*_ST_ values were also recalculated using the null allele correction in FreeNA. Genetic structure was also investigated with/without prior sample definition and a combination of admixture/no‐admixture models, using the model‐based Bayesian clustering programme STRUCTURE V2.3.4. (Pritchard *et al*., 2000). Simulations were run over 500,000 Markov chain Monte Carlo iterations with the first 100,000 repetitions discarded as burn‐in. Optimal *K* values (from a range of 1–5) were identified using the log probability of data (Pr(X/K)).

Individual assignment tests with Bayesian allele frequency estimation (Rannala & Mountain, 1997) were performed in GENECLASS 2 (Piry *et al*., 2004) to assess both the pattern and power with which individuals were assigned to species groups. Following Bekkevold *et al*. (2015) a two‐step approach was employed. In the first step, each individual was assigned to the species group from which its genotype had the largest probability of occurring (standard assignment). The second step aimed to disentangle cases where individuals were weakly assigned from “confident assignment” by imposing a more restrictive criterion wherein only assignments with probabilities >0.95 (following Hauser *et al*., 2006) were used. Self‐assignment tests using the leave one out method were first used to assess the power of the analysis and then assignment of individuals treated as of unknown origin were performed as directed by the other results.

## 3 RESULTS

### 3.1 mtDNA sequence data

**Figure 2:**
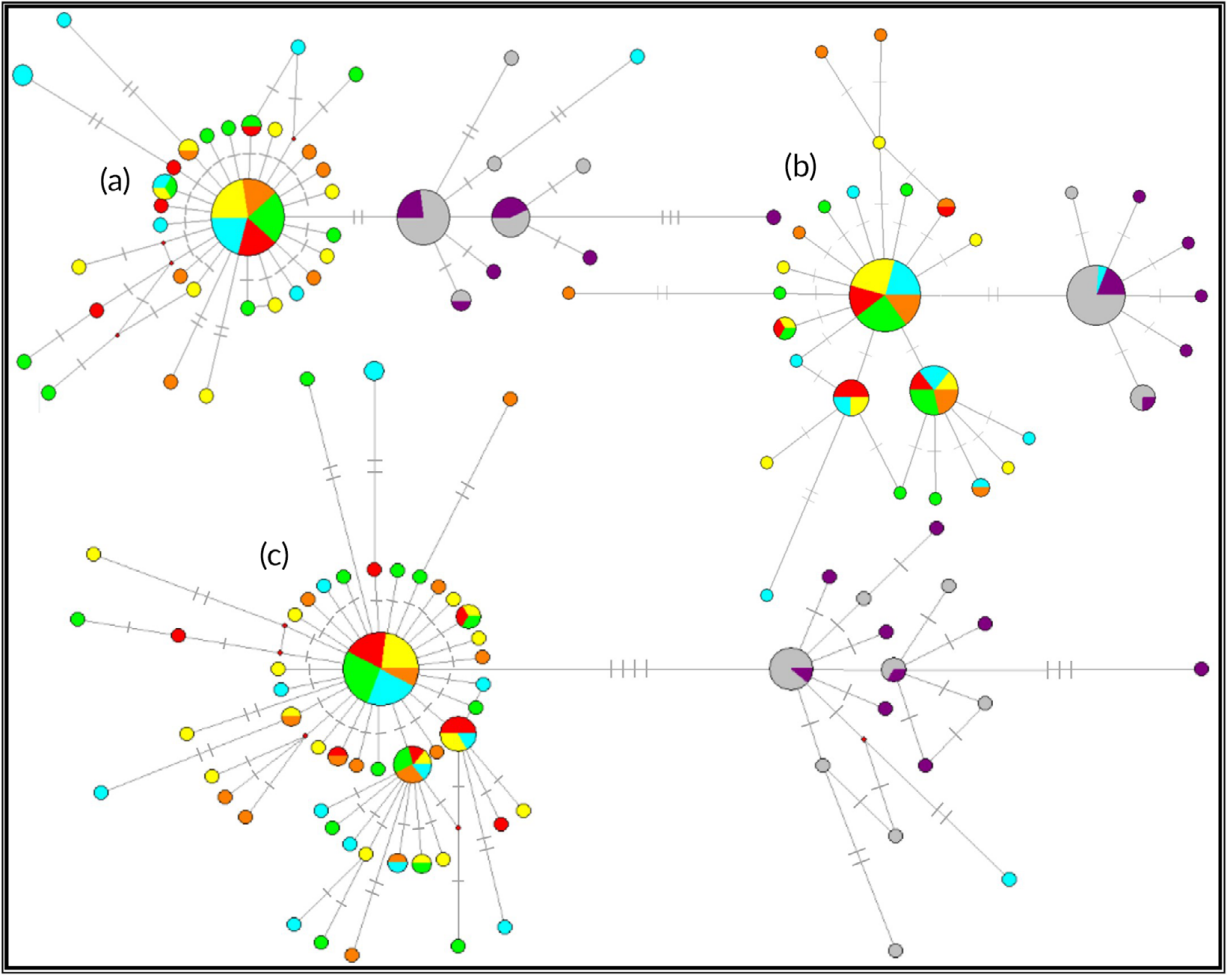
Median joining haplotype networks of *T. trachurus* and *T. capensis* based on (a) 605 bp of mtDNA COI, (b) 470 bp of mtDNA CR and (c) 1075 bp of concatenated sequence. Each dash represents a single site difference, node sizes are proportional to the overall haplotype frequency and pie discs correspond to within sample frequencies. 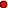, MH; 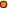, PO; 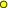, ME; 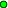, MT; 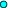; GT; 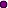, AN; 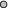, SAS

**Figure 3:**
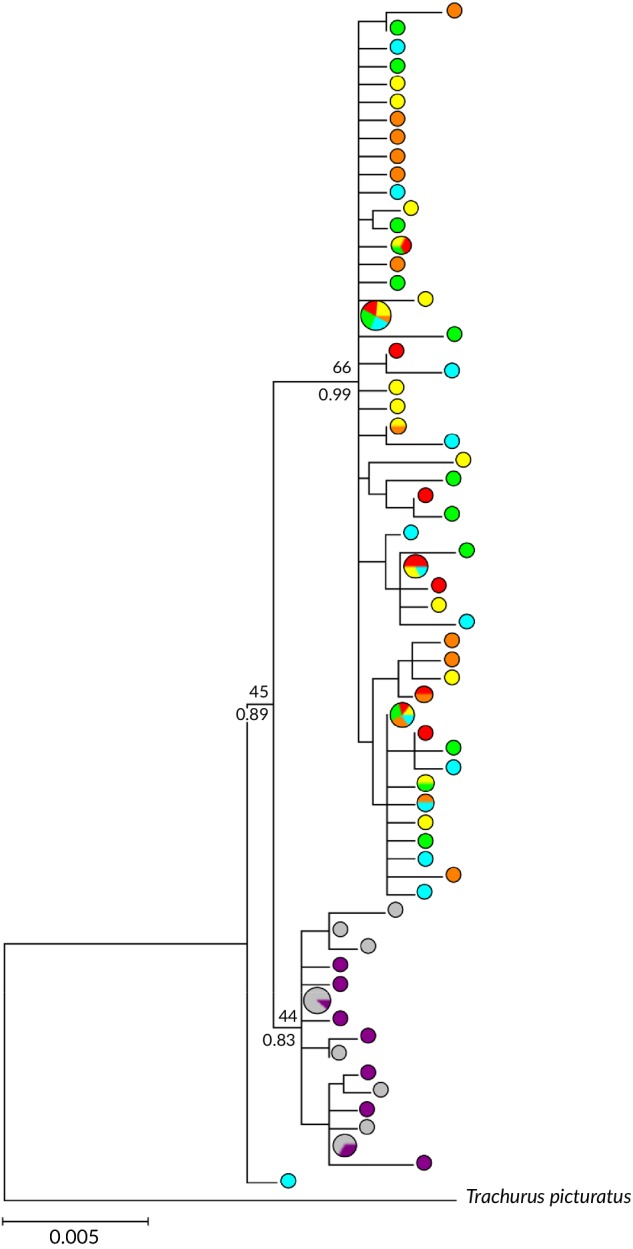
Maximum likelihood phylogeny of *T. trachurus* and *T. capensis* haplotypes resolved from 1075 bp of concatenated mtDNA COI and CR with bootstrap support given above branches. Statistical support for nodes from the corresponding Bayesian analyses (BI) are reported below branches. For clarity, nodal support is only reported where bootstrap support and/or BI probability were over 70% or 0.7, respectively. Pie charts denote the proportional representation of the associated haplotype among sites. 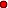, MH; 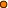, PO; 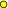, ME; 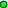, MT; 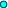, GT; 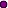, AN; 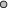, SAS

A 605 bp fragment of COI (*n* = 126 individuals) and 470 bp fragment of the CR (*n* = 125 individuals) region were aligned. Levels of diversity were extremely similar for both the COI and CR. The COI alignment revealed 46 polymorphic sites defining 41 haplotypes while for CR there were 33 variable sites and 31 haplotypes. Concatenated COI and CR sequences defined 66 haplotypes. The 66 haplotypes separated into two shallowly diverged reciprocally monophyletic clades (Figures 2 and 3). There was a clear spatial separation of these clades with them only co‐occurring in the Ghana sample. One was found across the NE Atlantic and Ghana (and was absent from Angola and South Africa). The other clade was found only in South Africa, Angola and a single Ghanaian individual (Figures 2 and 3). These clades are hereafter referred to as the *T. trachurus* and *T. capensis* clades, respectively, based on BLAST. Based on the concatenated sequences mean estimates of divergence time between the clades were 1.08 (95% probability, range 0.6–1.5) and 0.1 (95% probability, range 0.06–0.14) million years before present (BP), assuming 0.14% and 1.5% divergence rates, respectively.

Levels of intrasample variation were similar among all samples, as reported in Table 1. Pair‐wise tests (Table 2) indicated no significant differentiation within either species (only Ghana *T. trachurus* included here). Tajima's *D* and Fu's *F*s tests for neutrality were significant and negative for both clades. Mismatch distribution patterns conformed to demographic expansion models in all cases (COI, CR, concatenated sequence) except the CR‐based tests for *T. trachurus*. For concatenated sequences *T. capensis τ* = 0.615, leading to expansion time estimates of 38, 140 years BP and 408, 638 years BP, depending on the use of a 1.5% and 0.14% sequence divergence rate, respectively. For *T. trachurus* (*τ* = 0.6) expansion time estimates were 37, 209 years BP and 398, 671 years BP using the same divergence rates.

**Table 2:** Pair‐wise mtDNA Φ_ST_ values between samples

	PO	MH	ME	MT	GT	AN
MH	0.036					
ME	0.012	−0.024				
MT	0.005	−0.014	−0.012			
GT	0.010	−0.015	−0.006	−0.006		
AN	**0.619**	**0.655**	**0.636**	**0.610**	**0.557**	
SAS	**0.681**	**0.717**	**0.687**	**0.665**	**0.617**	−0.004

*Note*: Significant values denoted in bold. Sample codes as in Table 1.

Abbreviation: MT, Morocco: Tiznit.

### 3.2 Microsatellites

In total 10 loci were screened across 189 individuals and seven sample locations (Table 1). Allele sizes differed in expected multiples of the microsatellite repeat motifs. Thirty out of 70 locus by sample location comparisons deviated significantly from Hardy–Weinberg expectations (critical *P* = 0.05). Heterozygote deficits were less prominent for three loci, with the loci Tt48, TmurB116 and TmurC4 reporting significant test results for 0, 1 and 2 sample locations, respectively (Supporting Information Table S1). These three loci also reported the lowest average (across sample site) estimated null allele frequencies (NAF): (NAF)Tt48 = 0.008, (NAF)TmurB116 = 0.002 and (NAF)TmurC4 = 0.05). Excluding these three loci the average null allele frequency per locus ranged from 0.08 (TT113) to 0.26 (A115) with a mean of 0.14 (Supporting Information Table S2).

**Figure 4:**
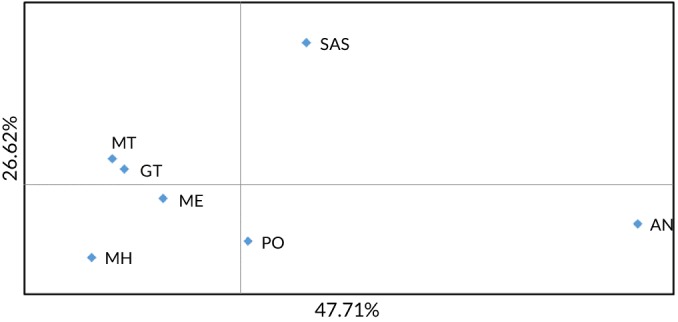
PCoA of pair‐wise nuclear *F*_ST_ values estimated from the three loci showing little evidence of null alleles. For abbreviations see Table 1

Based on the patterns of heterozygote deficits *F*_*ST*_‐based analyses were performed for three data sets: (a) all loci unedited; (b) all loci with null allele correction; and (c) the three loci with negligible heterozygote deficits. Across all samples (both species/clades) nuclear differentiation was highly significant (*F*_ST_ without null allele correction = 0.015, *F*_ST_ with null allele correction = 0.016, *F*_ST_ three loci subset = 0.015). Pairwise *F*_ST_ values for each of the three nuclear data sets supported the differentiation of the *T. capensis* (Angolan and South Africa) from the northern samples (*T. trachurus* except for that one individual in Ghana with *T. capensis* haplotype) (Figure 4). Patterns of pair‐wise *F*_ST_ among the northern samples (*i.e.*, Portugal to Ghana) differed slightly depending on the nuclear data set. For the three loci data set no significant pairwise *F*_ST_ values were obtained (Supporting Information Table S3). For the l0 loci data set with no null allele correction, the Ghana sample yielded significant *F*_ST_ values in all pairwise comparisons, whereas after null allele correction significant *F*_ST_ values were only obtained in comparisons between the Ghana sample and the Portugal and Morocco: Tiznit (MT) samples (Table 3).

**Table 3:** Pair‐wise *F*_ST_ values based on 10 microsatellite loci with statistically significant values in bold

	PO	MH	ME	MT	GT	AN	SAS
PO	–	−0.001	−0.001	0.001	**0.012**	**0.032**	**0.012**
MH	0.004	–	−0.001	0.002	0.005	**0.008**	**0.008**
ME	−0.003	−0.001	–	0.002	0.005	**0.015**	**0.012**
MT	0.005	0.006	0.002	–	**0.007**	**0.022**	**0.011**
GT	**0.026**	**0.010**	**0.011**	**0.014**	–	**0.017**	**0.018**
AN	**0.019**	**0.028**	**0.038**	**0.054**	**0.041**	–	**0.012**
SAS	**0.019**	**0.019**	**0.021**	**0.021**	**0.021**	**0.026**	–

*Note*: Sample codes as in Table 1. Above the diagonal are values obtained after correction for null alleles using the ENA method. Below the diagonal are values from uncorrected data.

Abbreviation: MT, Morocco: Tiznit.

STRUCTURE analyses performed for the unedited and three loci data sets all failed to resolve any structure, with *K* = 1 unanimously supported. The lowest *P* for *K* = 1 was 0.89 for the analysis performed with the three loci subset and using LocPrior and assuming no admixture. Classical assignment tests differ from STRUCTURE in that they require some *a priori* classification of baseline samples for individuals to be assigned to. We were particularly interested in assessing whether the *T. capensis* mitotype bearing individual was assigned to either *T. trachurus* or *T. capensis*. To assess the power of the analysis we first performed species self‐classification tests wherein we set up a “pure” *T. trachurus* baseline group by pooling the Portuguese (PO) and Moroccan (MH, ME and MT) samples and a “pure” *T. capensis* baseline group by pooling the Angolan (AN) and South African (SAS) samples. For the standard assignment with unedited data 72% (*n* = 101) individuals correctly self‐assigned to these pure species groups (82 out of 108 for *T. trachurus* and 19 out of 32 for *T. capensis*). Applying the criteria for confident assignment reduced these numbers to only 61 *T. trachurus* and 10 *T. capensis* being correctly self‐assigned. Standard assignment with the three‐locus data set revealed only 64% (*n* = 90) of individuals correctly self‐assigned to species (74 *T. trachurus* and 16 *T. capensis*). On applying the more restrictive criterion for confident assignment, only 13 *T. trachurus* and no *T. capensis* correctly self‐assigned. For the full data set the Ghanaian individual confidently assigned to *T. trachurus* (*P* = 0.99), but for the three‐loci data set it only assigned to *T. trachurus* with a probability of 0.57 (*T. capensis* membership probability = 0.43).

## 4 DISCUSSION

### 4.1 Divergence between the species

A salient feature of the results was the resolution of two reciprocally monophyletic clades that exhibited a clear spatial partitioning. One clade was found among the NE Atlantic, Mediterranean and Ghana samples and was absent from Angola and South Africa, and is hereafter referred to as the *T. trachurus* clade. The other clade was found only in South Africa, Angola and a single Ghanaian individual and is hereafter referred to as the *T. capensis* clade. BLAST results support these clade designations. Ghana was the only location where both clades were detected. There was no significant spatial structuring within either clade. Microsatellite genotypes were analysed to provide a nuclear perspective and revealed a large number of heterozygote deficits. Strong heterozygote deficits have been reported for many fish species and can be due to biological factors, such as selection, inbreeding and Wahlund effects, and/or technical artefacts, most commonly null alleles (Hoarau *et al*., 2002). The association of the heterozygote deficits with certain loci and not others in this study would suggest a prominent role for null alleles. In their population genetic study of *T. trachurus* Kasapidis & Magoulas (2008) reported signals compatible with spatially variable null allele effects at three out of the four loci developed from *T. trachurus* and employed here. This study also used six microsatellite loci developed from *T. murphyi* sequences and such cross‐species amplification increases the likelihood of null alleles (*e.g.*, Panova *et al*., 2006). To account for possible errors stemming from null alleles additional analyses were performed for null allele corrected genotypes and omitting loci with null alleles. A replicated pattern in *F*_*ST*_‐based analyses across the data sets was the separation of the Angola and South Africa samples from the remaining samples. This aligns with the distribution of the *T. capensis* and *T. trachurus* clades and supports the broad‐scale nuclear differentiation between the species.

The shallow divergence between the *T. trachurus* and *T. capensis* mtDNA clades fits with an overall pattern of low levels of interspecific genetic divergence within *Trachurus* as reported by Cardenas *et al*. (2005). Such low levels of DNA divergence have been linked to a combination of recent divergence and low nucleotide substitution rates. Both Bektas & Belduz (2008) and Cardenas *et al*. (2005) also suggested similar low mutation rates for both coding and non‐coding mtDNA regions, a feature evident in the near identical levels of variation at both COI and CR here. Estimates of divergence between the *T. trachurus* and *T. capensis* mtDNA clades fitted within the broad timescales inferred by Cardenas *et al*. (2005), who suggested the divergence occurred sometime after 2.8 or 2.3 MYA depending on the divergence rate used. While the IMA‐based method here has been shown to perform well in cases of shallow divergences (McKeown *et al*., 2019), a fundamental consideration for any such estimates is that commonly inferred and even fossil obtained “species” level mutation rates may be considerably lower than those experienced at intraspecific levels and/or over more recent evolutionary time frames (1–2 MYA). This can lead to an over‐estimation of the ages of divergence and demographic events (Grant, 2015; Ho *et al*., 2005, 2007; Hoareau, 2015). Such concerns have led investigators to apply 2–10‐fold rate corrections (Gonzalez‐Wevar *et al*., 2011; McKeown *et al*., 2019; Pardo‐Gandarillas *et al*., 2018) to estimates of demographic events. While mindful of both general and *Trachurus* specific rate considerations the data are compatible with divergence between of *T. trachurus* and *T. capensis* clades during the Pleistocene, as has been observed for a number of other marine taxa (Burridge, 2002; Grant *et al*. 2005; Ludt *et al*., 2015; Stepien & Rosenblat 1996). Applying similar rate adjustments to the dating of expansion events indicated by mismatch analysis and neutrality tests suggests that such expansions have occurred within the postglacial period, a timeframe which fits with the general view put forward by Grant (2015). In conclusion, divergence of the clades occurred during the glacial period and both lineages exhibit signals of postglacial expansions.

### 4.2 Secondary contact in Ghana

An interesting finding was the co‐occurrence of both mtDNA clades in the Ghana sample with a single individual bearing the *T. capensis* clade. Secondary contact between divergent mtDNA lineages occurs in a number of fish taxa and individual assignment‐based analyses have resolved cases where such secondary contact is associated with hybridization (*e.g.*, Sala‐Bozano *et al*., 2009) or mechanical mixing without interbreeding (Healey *et al*., 2018a). In assignment tests using all loci the Ghana individual bearing the *T. capensis* clade assigned with high probability to the *T. trachurus* group, suggesting this individual reflects recent or ancestral gene flow between the species rather than a *T. capensis* migrant. However, when assignment tests were performed for the subset of loci without limited null allele signatures this individual had a similar probability of assigning to either species group. This discrepancy, alongside the potential confounding effects of null alleles and overall low level of correct self‐assignment to species, indicates that the data conferred little power to individual assignment tests. More powerful genomic assays are needed to robustly distinguish between mixing and hybridization. If there is a high level of introgression between both species within the region the Ghana sample would be expected to be genetically intermediate. However, PCoA of pair‐wise *F*_ST_ scores clearly showed it to cluster with the *T. trachurus* samples distinct from the *T. capensis* samples, suggestive of low rates of hybridization.

### 4.3 Population structure within both species

There was no significant mtDNA differentiation between the *T. capensis* samples from Angola and South Africa. This is a striking result in light of the high level of mtDNA divergence, including examples of reciprocal monophyly, between Angolan and South African sites for a number of marine taxa due to the BUS biogeographic boundary (Gwilliam *et al*., 2018; Henriques *et al*., 2012, 2014; Reid *et al*., 2016). This could be due to a combination of the previously mentioned slow mtDNA mutation rates for *Trachurus* as well as effective gene flow across the BUS as might be expected based on the species' life history. Specifically, while the cool upwelled waters of the BUS are a dispersal barrier for many species, *T. capensis* is an upwelling associated species with peak abundance observed across the main upwelling regions and is expected to freely disperse across the BUS (Axelsen *et al*., 2004; Barange *et al*., 1998; McLaverty, 2012). Microsatellites did reveal a significant *F*_*ST*_ value between the Angolan and SAF samples. However, in light of the small sample size for Angola and prevalence of null alleles the biological validity of this differentiation should be considered questionable unless confirmed by other studies (Knutsen *et al*., 2011). Overall, the strongest elements of the data and life history expectations suggest connectivity between Angolan and South African *T. capensis*. The current management of the species as distinct northern and southern BUS stocks therefore represents a potential misalignment between management and population boundaries (Reiss *et al*. 2009).

mtDNA and microsatellites revealed no significant genetic structuring among the NE Atlantic and Mediterranean *T. trachurus* samples. This aligns with the previous *T. trachurus* study by Kasapidis & Magoulas (2008), which found no significant structure across a more extensive sampling of NE Atlantic and Mediterranean waters using four of the microsatellites employed here. Genetic cohesion between Atlantic and Mediterranean samples is also described for other horse mackerel species and indicates that the Almeria‐Oran frontal system is not a prominent phylogeographic break in this group (Comesana *et al*., 2008; Moreira *et al*., 2019). Nuclear *F*_ST_ tests performed for both the null allele corrected and the subset of HWE conforming loci and mtDNA Φ_ST_ broadly supported a lack of differentiation of the Ghana sample from the other *T. trachurus* samples. Although there was a gap in our sampling between Ghana and Europe, high gene flow between European and north‐west African sites is also suggested for the blue jack mackerel (*T. picturatus*) (Moreira *et al*., 2019). The available data therefore indicate that *T. trachurus* exhibits high gene flow and an absence of genetic structure across most of its sampled range to date.

An important consideration previously highlighted by Kasapidis & Magoulas (2008) is that the absence of genetic structure in *T. trachurus* and *T. capensis* does not mean there is no significant isolation of stocks on timescales of interest to management (Hauser & Carvalho, 2008). Resolution of such stock structure will require more powerful genomic approaches, such as RADseq, which offer the potential to genotype large numbers of loci and identify markers under selection (Gagnaire *et al*., 2015; Mullins *et al*., 2018). Combining genomic approaches with information from ontogenetic markers could also provide synergistic insights. Ontogenetic markers have already proven highly informative in mackerel with studies of parasite assemblages (MacKenzie *et al*., 2008), morphometrics (Murta *et al*., 2008; Stransky *et al*., 2008) and life history traits (Abaunza *et al*., 2008b) reporting finer scale population heterogeneity than indicated by population genetic methods to date.

Despite signatures of historical population size fluctuations all samples exhibited similar levels of genetic variation that were comparable to levels in other small pelagic fish species (*Sardinella aurita*: Tringali & Wilson, 1993; *Engraulis encrasicolous*: Silva *et al*., 2017; *Engraulis japonicus*: Liu *et al*. 2006; and *T. picturatus*: Moreira *et al*., 2019). Although fisheries induced loss of genetic variation, as observed in other taxa (Cimmaruta *et al*., 2005), cannot be discounted the data indicate that local populations retain high levels of genetic variation and if current effective population sizes are maintained genetic drift alone will be insufficient to erode variation.

## 5 CONCLUSIONS

The results of this study both inform conservation management strategies and direct future research. The data support the view that *T. trachurus* is not found in Angolan and South African waters as has sometimes been suggested. The co‐occurrence of both species' clades in Ghana also reveals this area to be an apparent suture zone between their ranges. Further sampling of the region is required to assess the extent of spatial overlap between both species. The results here suggest both species are likely being indiscriminately harvested within this region, which may severely compromise stock sustainability (Healey *et al*., 2018a,b; McKeown *et al*., 2015). More powerful genomic‐based assays will also be useful to permit individual‐based analyses of hybridization and fish traceability (Helyar *et al*., 2011; Hemmer‐Hansen *et al*., 2019). Such genomic assays will also be necessary for both species to align spatial management units with patterns of stock recruitment. Until such information is available the current designation of regional management units in *T. trachurus* should be maintained as a precautionary measure. Paradoxically, for *T. capensis* the likelihood of connectivity between the northern and southern BUS operational stocks should be considered in attempts to understand potentially cohesive stock dynamics (Frisk *et al*., 2008).

## AUTHOR CONTRIBUTIONS

AJEH and MFF performed fieldwork with additional samples provided by FKEN and WMP. AJEH and NJMK performed analysis and prepared the manuscript. All authors commented on the manuscript.

## Supporting information


**Supporting Information Table S1.** *P* values from single locus by sample tests of conformance to Hardy–Weinberg equilibrium genotype proportions

**Supporting Information Table S2.** Estimated frequency of null alleles for each locus × sample combination

**Supporting Information Table S3.** Pairwise *F*_ST_ estimated from the three loci conforming to HWE, *i.e.*, no null alleles (below diagonal). Reported corresponding *F*_ST_ *P* values estimated following permutation (above diagonal).
