# Supplementary material for: Genetic analysis provides insights into species distribution and population structure in East Atlantic horse mackerel (Trachurus trachurus and T. capensis)
Source: J Fish Biol. 2020 Feb 20;96(3):795–805. doi: 10.1111/jfb.14276 (PMC7079130; doi:10.1111/jfb.14276)
Supplement: Supplementary file 1 — Supporting Information Table S1. P values from single locus by sample tests of conformance to Hardy–Weinberg equilibrium genotype proportions [file JFB-96-795-s001.docx]

Supplementary Table 1. P values from single locus by sample tests of conformance to Hardy-Weinberg equilibrium genotype proportions.

| Locus | PO | MH | ME | MT | GT | AN | SAS |
| --- | --- | --- | --- | --- | --- | --- | --- |
| TmurA101 | 0.021 | 0.060 | 0.000 | 0.000 | 0.000 | 0.132 | 0.057 |
| TmurA104 | 0.254 | 0.930 | 0.000 | 0.081 | 0.030 | 0.488 | 0.000 |
| TmurA115 | 0.003 | 0.000 | 0.000 | 0.000 | 0.000 | 0.169 | 0.003 |
| TmurB104 | 0.015 | 0.172 | 0.000 | 0.067 | 0.099 | 0.886 | 0.256 |
| TmurB116 | 0.199 | 0.058 | 0.934 | 0.001 | 0.599 | 0.484 | 0.962 |
| TmurC4 | 0.001 | 0.492 | 0.852 | 0.099 | 0.196 | 0.626 | 0.005 |
| TT48 | 1.000 | 0.958 | 0.167 | 0.953 | 0.079 | 0.659 | 0.787 |
| TT62 | 0.387 | 0.698 | 0.001 | 0.115 | 0.000 | 0.141 | 0.013 |
| TT113 | 0.008 | 0.129 | 0.044 | 0.468 | 0.000 | 0.559 | 0.027 |
| TT29 | 0.204 | 0.027 | 0.000 | 0.001 | 0.000 | 0.720 | 0.005 |
