# Supplementary material for: Genetic analysis provides insights into species distribution and population structure in East Atlantic horse mackerel (Trachurus trachurus and T. capensis)
Source: J Fish Biol. 2020 Feb 20;96(3):795–805. doi: 10.1111/jfb.14276 (PMC7079130; doi:10.1111/jfb.14276)
Supplement: Supplementary file 2 — Supporting Information Table S2. Estimated frequency of null alleles for each locus × sample combination [file JFB-96-795-s002.docx]

Supplementary Table 2. Estimated frequency of null alleles for each locus x sample combination.

|  | TmurA101 | TmurA104 | TnurA115 | TmurB104 | TmurB116 | TmurC4 | TT48 | TT62 | TT113 | TT29 |
| --- | --- | --- | --- | --- | --- | --- | --- | --- | --- | --- |
| PO | 0.17 | 0.14 | 0.29 | 0.23 | 0.00 | 0.11 | 0.00 | 0.08 | 0.19 | 0.07 |
| MH | 0.23 | 0.00 | 0.38 | 0.20 | 0.05 | 0.14 | 0.00 | 0.00 | 0.10 | 0.19 |
| ME | 0.23 | 0.04 | 0.32 | 0.12 | 0.03 | 0.00 | 0.03 | 0.16 | 0.07 | 0.24 |
| MT | 0.19 | 0.05 | 0.33 | 0.15 | 0.04 | 0.07 | 0.02 | 0.14 | 0.05 | 0.13 |
| GT | 0.13 | 0.12 | 0.22 | 0.09 | 0.02 | 0.02 | 0.02 | 0.19 | 0.10 | 0.21 |
| AN | 0.07 | 0.10 | 0.13 | 0.00 | 0.00 | 0.00 | 0.00 | 0.00 | 0.00 | 0.00 |
| SAS | 0.11 | 0.16 | 0.18 | 0.14 | 0.00 | 0.03 | 0.00 | 0.09 | 0.06 | 0.17 |
