# Supplementary material for: Genetic analysis provides insights into species distribution and population structure in East Atlantic horse mackerel (Trachurus trachurus and T. capensis)
Source: J Fish Biol. 2020 Feb 20;96(3):795–805. doi: 10.1111/jfb.14276 (PMC7079130; doi:10.1111/jfb.14276)
Supplement: Supplementary file 3 — Supporting Information Table S3. Pairwise F ST estimated from the three loci conforming to HWE, i.e., no null alleles (below diagonal). Reported corresponding F ST P values estimated following permutation (above diagonal). [file JFB-96-795-s003.docx]

Supplementary Table 3. Pairwise F_ST_ estimated from the three loci conforming to HWE i.e. no null alleles (below diagonal). Above diagonal are reported corresponding F_ST_ P values estimated following permutation

|  | PO | MH | ME | MT | GT | AN | SAS |
| --- | --- | --- | --- | --- | --- | --- | --- |
| PO | - | 0.312 | 0.469 | 0.362 | 0.282 | 0.016 | 0.005 |
| MH | 0.028 | - | 0.644 | 0.190 | 0.255 | 0.156 | 0.029 |
| ME | 0.013 | 0.012 | - | 0.223 | 0.545 | 0.004 | 0.002 |
| MT | 0.016 | 0.022 | 0.009 | - | 0.498 | 0.004 | 0.008 |
| GT | 0.016 | 0.018 | 0.005 | 0.006 | - | 0.003 | 0.005 |
| AN | 0.061 | 0.043 | 0.046 | 0.057 | 0.055 | - | 0.014 |
| SAS | 0.047 | 0.038 | 0.025 | 0.023 | 0.023 | 0.047 | - |
